# β­caryophyllene oxide induces apoptosis and inhibits proliferation of A549 lung cancer cells

**DOI:** 10.1007/s12032-023-02022-9

**Published:** 2023-05-26

**Authors:** Sameh M. Shabana, Nahla S. Gad, Azza I. Othman, Aly Fahmy Mohamed, Mohamed Amr El-Missiry

**Affiliations:** 1 Department of Zoology, Faculty of Science, Mansoura University, Mansoura, 35516 Egypt; 2 The International Center for Advanced Researches (ICTAR-Egypt), Al-Azhar University, Cairo, 307422 Egypt

**Keywords:** Lung cancer, Essential oils, β-caryophyllene oxide, Apoptosis, Cell cycle arrest, Oxidative stress

## Abstract

One of the most common cancers that result in death is lung cancer. There is new hope in the fight against lung cancer thanks to the chemopreventive properties of natural dietary substances like β-caryophyllene oxide (CPO), and research is currently being done to test this theory. CPO, a sesquiterpene isolated from medicinal plant essential oils, inhibits carcinogenesis and has been effective in treating many cancers. This study examined how CPO affected proliferation of human lung cancer A549 cells. CPO was found to have an inhibitory concentration (IC50) of 124.1 g/ml. The proliferative markers Ki67 and PCNA were significantly inhibited after cells were treated with CPO at a concentration of 50 g/ml compared to controls. CPO-treated cells expressed more P21, P53, and DNA strand breaks than controls. This was accompanied by a significant cell cycle arrest in the S and G2/M phases. In treated A549 cells, this was also associated with a significant induction of apoptosis, as shown by the upregulation of the expression of caspases 3, 7, and 9, as well as Bax, and the downregulation of Bcl-2. Furthermore, the redox status of treated A549 cells revealed a marked rise in GSH and GPx activity levels and a decline in 4-HNE levels, indicating low oxidative stress following CPO treatment of A549 cells. In conclusion, cell cycle arrest and apoptosis, which are unrelated to oxidative stress, were the mechanisms by which CPO reduced cancer lung cell growth. This finding might be a potential therapeutic target for the treatment of lung cancer.

**Graphical abstract:**

Hypothetical scheme of CPO anticancer effects (mechanism of signaling) in A549 cells; in vitro. CPO treatment increases expression of p21, p53 and DNA fragmentation. These events cause arrest of cell cycle which was associated with significant induction in apoptosis via increase expression of caspases (-3,-7,-9), and Bax and downregulation of Bcl-2.

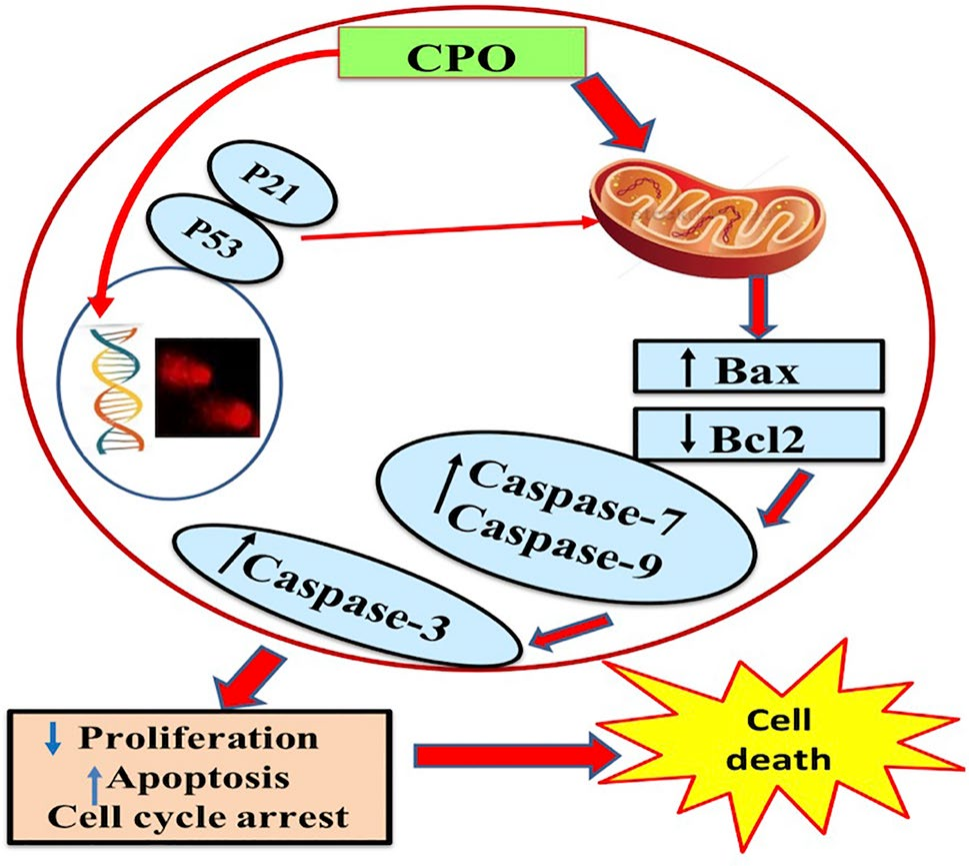

## Introduction

Lung cancer, which is thought to be the primary cause of cancer deaths worldwide, has the fourth-highest incidence of all malignancies [1]. Lung cancer was the most common type of cancer that caused deaths in 2020 [2]. Small cell lung cancer, which is less common, and non-small cell lung cancer (NSCLC), which accounts for the majority of lung cancer cases, are the two main types of lung cancer [3]. These studies confirm the requirement for the future development of innovative, secure, and efficient chemotherapeutic drugs.

Chemotherapy primarily targets cancer cells' oxidative damage, apoptosis, and inhibition of proliferation by inducing cell cycle arrest in order to prevent and treat cancer [4]. Due to a lack of conclusive research findings, there is currently a heated debate about the use of antioxidants in cancer therapy [5]. Antioxidant therapy may be quite effective in some cancer cases, but it may not have an impact on the growth of cancer cells in others [6]. This necessitates further research into this mystery. One of the many variables that have been studied in the emergence of cancer is oxidative stress. The excessive production of free radicals in tissues and cells that increases the risk of tumour initiation and progression is the root cause [7]. Enhanced cell survival and proliferation, DNA damage, and genetic instability have all been linked to higher levels of reactive oxygen species (ROS) production, according to reports [8]. However, it has also been noted that higher levels of ROS promote anti-tumorigenic signalling by promoting oxidative stress-induced tumour cell death. Tumor cells have developed a system that allows them to express higher levels of antioxidant proteins for detoxification while maintaining pro-tumorigenic signalling and resistance to apoptosis in order to deal with excessive ROS production [9]. Because reducing oxidative stress with potent antioxidants contributes significantly to characteristics of cancer like angiogenesis, invasiveness, stemness, and the ability to metastasize, it has been used as a critical strategy for cancer prevention [10]. The presence of a diverse class of polyphenols in β-caryophyllene oxide (CPO) has been demonstrated to exert strong antioxidant effects with an anticancer impact [11]. A potential cancer treatment involves ROS modulation by CPO because cancer cells have an altered redox equilibrium.

For a very long time, medicinal plants have been used in healthcare. Many natural products derived from plants have undergone extensive testing for their anticancer efficacy over the past few decades. This suggests that using medicinal plants to treat cancer may be promising. The use of medicinal plants as complementary therapies for various cancers has grown recently throughout the world. Essential oils, whose primary constituents are monoterpenes and sesquiterpenes, including CPO, are among the most important active metabolites of medicinal plants [12]. One of the main active ingredients in essential oils, CPO can be obtained from a variety of spices and is present in significant amounts in a number of edible plants. Additionally, CPO has a number of biological effects, including analgesic, antimicrobial, anticarcinogenic, anti-inflammatory, and antimicrobial properties [13]. According to several studies, CPO has anticancer properties for various cancer cell lines [11, 14, 15]] and can boost the effectiveness of a number of anticancer medications [14, 16]. Human gingival fibroblasts and human oral mucosa epithelial cells are two examples of normal cells that are unaffected by CPO, making it a safe substance [17].

This study evaluated apoptosis, apoptotic regulating proteins, cell cycle arrest, DNA integrity, and redox balance to investigate the anti-proliferative mechanisms of CPO on NSCLC cells, i.e., A549 cells.

## Materials and methods

### Chemicals

CPO was purchased from Sigma-Aldrich Chemical Company (Gillingham, United Kingdom) and prepared at 50 μg/ml in dimethyl sulfoxide (DMSO 0.3%), which was purchased from ICI-UK. Local suppliers provided additional lab chemicals.

### Cell culture

The cell culture division at VACSERA in Egypt provided the A549 human lung cancer cell line. A549 cells in the form of frozen ampoules bearing the reference number HTB-22 were imported from the American Type Culture Collection. Gibco® provided the RPMI-1640, foetal bovine serum (FBS), and penicillin/streptomycin (Invitrogen, Grand Island, NY, USA). A549 cells were grown in RPMI-1640 containing 10% FBS, 100 units/mL of penicillin/streptomycin, and 37 °C in an incubator with 95% humidity and 5% CO_2_. To maintain exponential growth, the cells were isolated using trypsin–EDTA and passaged every two to three days.

### Cytotoxicity test

A 3-(4,5-dimethylthiazol-2-yl)-2,5-diphenyl tetrazolium bromide (MTT) assay was used to evaluate cytotoxicity, as previously described [18]. Cell viability after CPO treatment was determined using the MTT assay. Initially seeded in 96-well plates (TPP, Swiss), cells (2 × 10^5^ cells/ml) were then exposed to twofold serially diluted concentrations of CPO (up to 4000 g/ml). An inverted microscope was used to find the cytotoxic effect (Hund, Germany). The media containing the drugs were taken out after 24 h and medium containing MTT stain (Sigma, M5655-1G; 0.5 mg/ml) was added. The formed formazan crystals were solubilized with DMSO after four hours of incubation at 37 °C. A microplate reader, the ELx 800, was used to measure the absorbance at each well at 570 nm (Biotek, Winooski, USA).

### Evaluation of DNA damage

Evaluation of DNA damage was performed using single-cell gel electrophoresis [19] on control and CPO-treated A549 cells. To achieve a density of 2.5 × 10^4^/ml, cells were combined in a 1:1 ratio with 1% low melting point agarose after being washed twice with phosphate buffered saline (PBS). The slides were then covered with a thin layer of 300 µl of cells in agarose. The slides were electrophoresed at 25 V for 30 min after being incubated for one hour in lysis buffer (60 mmol/l NaOH, 1 mol/l NaCl, 0.5% (w/v) N-lauryl sarcosine, pH 12.5) and another hour in DNA unwinding solution (40 mmol/l NaOH, 2 mmol/l EDTA, pH 13). Comets were stained with ethidium bromide and then examined with fluorescence inverted microscopy (Olympus CKX41) at a magnification of 40 × while using a green filter (Excitation filter BP480-550C). The pictures were then taken with a C-mount camera (Optika pro5 CCD camera). CASP software was used to directly calculate the percentage of the tail moment, the amount of DNA in the tail, and the length of the tail when quantifying DNA damage in the obtained images.

### Flow cytometric analysis of the cell cycle

A FACS Caliber Flow Cytometer (USA, CA, Sunnyvale, Becton Dickinson) that features a small, air-cooled, low-ion laser beam (488 nm) with 15 mW argon was used to conduct the flow cytometry. Cell cycle analysis was carried out as previously mentioned [20]. The cells were incubated in a solution (200 L) containing 200 g/ml of RNase A (Invitrogen Biotechnology, Carlsbad, CA, USA), 20 g/ml of propidium iodide (PI) (Sigma, St. Louis, MO, USA), and 0.1% Triton v/v in PBS after the sample was prepared. The samples were subjected to flow cytometric analysis following a 30-min dark incubation period. A percentage of cells in the G2/M, G0/G1, or S phases is displayed as a result.

### Apoptosis detection

With the aid of an Annexin V-FITC Apoptosis Detection Kit and a BD FACS Calibur Flow Cytometer (BD Biosciences, CA, USA), lung cancer cells' apoptosis was discovered (Biovision, USA). The emission wavelengths used to gather data were 530 nm for fluorescein isothiocyanate (FITC) and 670 nm for PI. The argon laser's excitation wavelength was 488 nm. Glutathione peroxidase (GPx) and glutathione (GSH) were measured using ELISA analysis, as well as 4-hydroxynonenal (4-HNE) and proliferation markers KI67 and PCNA. GSH, GPx, Ki-67, and PCNA were quantified using a quantitative sandwich immunoassay. Using Biodiagnostic's kits, the concentrations of GSH and GPx activities were estimated (Giza, Egypt). Ki-67 and PCNA were measured using rat Ki-67 ELISA Kits from Biorbyt in the United Kingdom (cat. no. orb410642) and rat PCNA ELISA Kits from MyBioSource.com in California (cat. no. MBS2515480), respectively. According to the manufacturer's instructions (FineTest, Wuhan, China), lipid peroxidation was calculated by estimating the concentration of 4-HNE using a competitive inhibition enzyme immunoassay technique with a detection range of (31.25–2,000 pg/ml). ELISA analysis software was used to measure all markers.

### Flowcytometric analysis of p53, p21, caspases (3, 7, and 9), Bax, and B-cl2

Santa Cruz Biotechnology provided the p53 (cat. no. sc-7480), p21 (cat. no. sc-6246), caspase-3 (cat. no. sc-271759), caspase-7 (cat. no. sc-56063), caspase-9 (cat. no. sc-56076), Bcl-2 (cat. no. sc-7382), and Bax (cat. no. sc-7480) (Santa Cruz, CA, USA). The right way to prepare cells was done so in accordance with the manufacturer's instructions. PBS/BSA buffer (1% BSA) was used to bring the cell suspension's concentration to 1 × 10^6^ cells/ml. Cell suspension was pipetted into test tubes. Fluorescein (FITC) conjugated antibodies that were appropriately labelled were added to the recommended dilution, thoroughly mixed, and incubated at room temperature for 30 min. After centrifuging the cells at 400 g for 5 min after being washed with 2 ml of PBS/BSA, the supernatant was discarded. Cells were suspended in 0.2 ml of either 0.5% paraformaldehyde in PBS/BSA or 0.2 ml of PBS/BSA. Data was collected using flow cytometry (Becton Dickinson, CA, USA). Cell Quest software (Accuri C6) was used to collect a total of 20,000 cells for analysis, and a histogram plot of conjugate fluorescence (x-axis) versus counts (y-axis) was made in logarithmic fluorescence intensity.

### Statistical analysis

The one-way ANOVA POST HOC (Tukey's and Dunnett's) test was used in the statistical analysis, which was carried out using the GraphPad Prism software. Results were presented as the mean ± standard deviation (SD) from three independent experiments, and statistical significance was determined by P values of < 0.05 or < 0.001.

## Results

### CPO inhibited cell proliferation and the proliferative markers

**Fig. 1:**
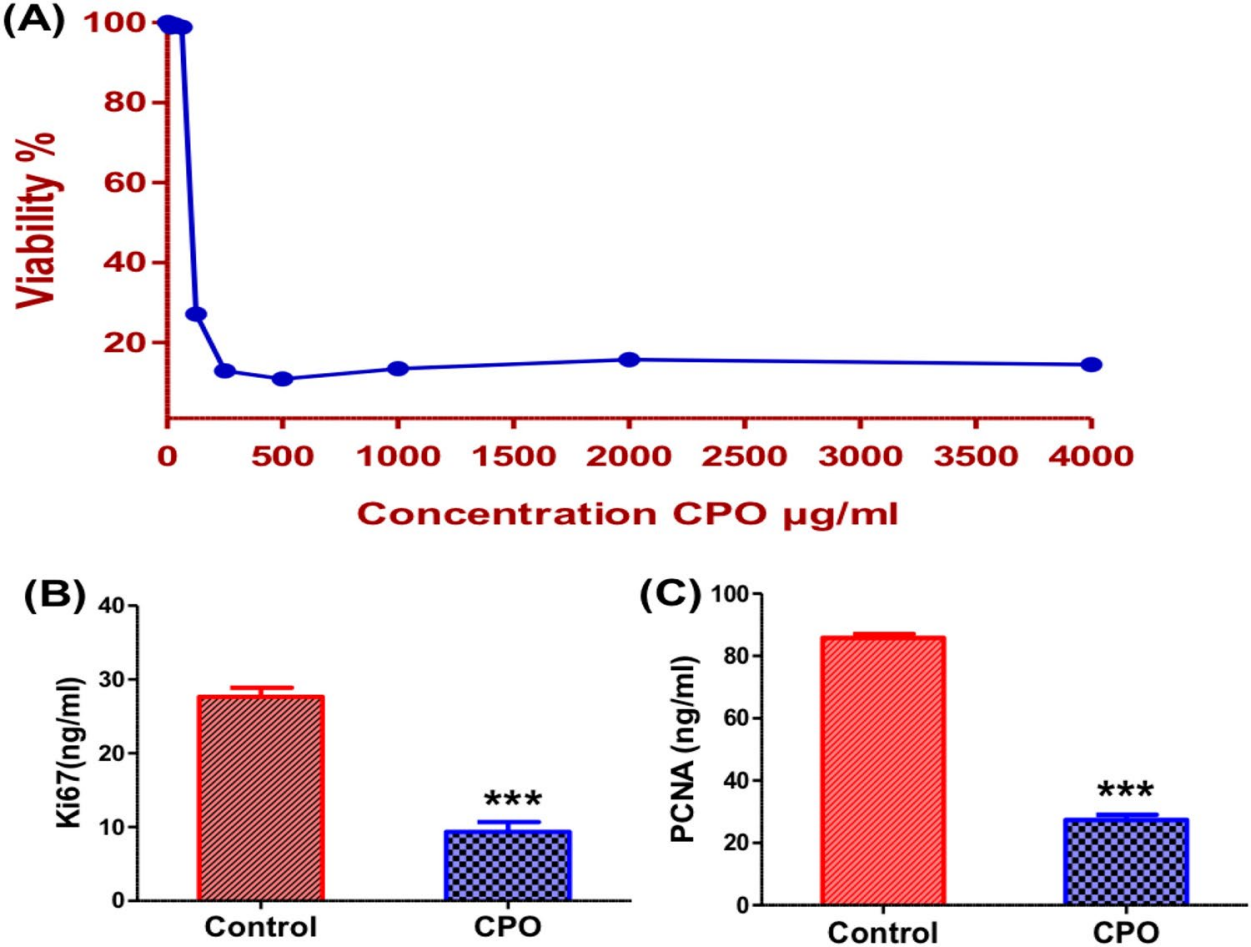
Cytotoxic and antiproliferative effects of CPO: **A** Cytotoxic effects of CPO in various concentrations after 24 h incubation with the A549 lung cancer cell line. Levels of Ki-67 (**B**) and PCNA (**C**) expression in the A549 lung cancer cell line after 24 h. The results are expressed as the mean ± SD values from at least three independent experiments. ***P < 0.001 for the CPO (50 µg/ml) treated group in comparison to the control group. CPO: β­caryophyllene oxide

A MTT assay was used to evaluate the inhibitory effects of CPO on the proliferation of A549 cells. As shown in (Fig. 1A), the mean viability percentage of CPO-treated cells decreased in a dose-dependent manner compared to control cells. The IC50 value of CPO was 124.1 µg/ml after 24 h of treatment. According to ELISA analysis, CPO-treated A549 cells showed a significant decrease in expression levels in both proliferative markers Ki-67 (P < 0.001; Fig. 1B) and PCNA (P < 0.001; Fig. 1C) compared to the control group.

### CPO repressed cell cycle progression in A549 cells

**Fig. 2:**
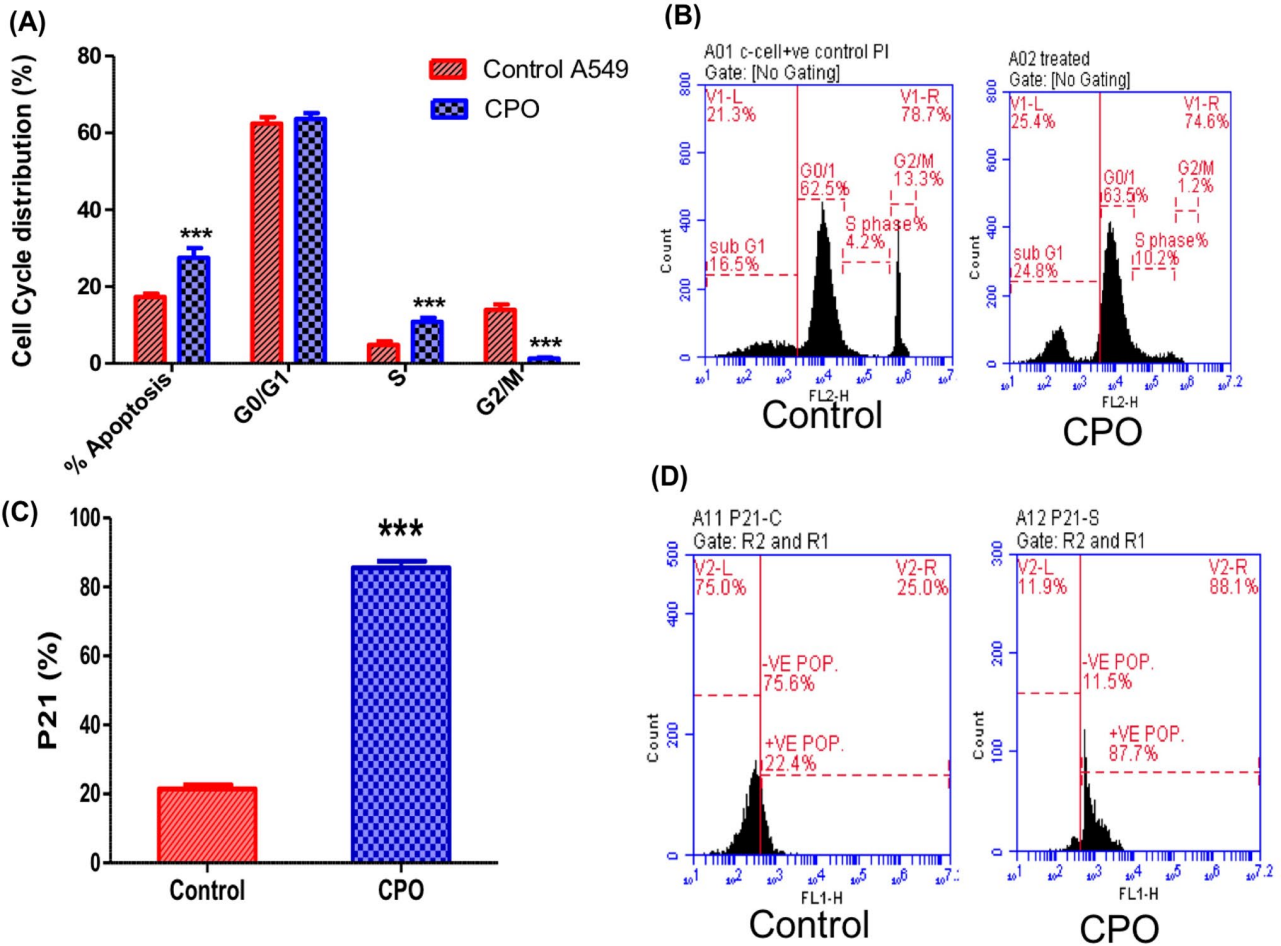
Cell cycle analysis and cell cycle inhibitor p21 expression in the A549 lung cancer cell line after 24 h, measured by flow cytometry. Quantitative analysis (**A**) and representative flow cytometric histogram (**B**) of the effect of CPO (50 µg/ml) on cell cycle progression in A549 cells. Quantitative analysis (**C**) and representative flow cytometric histogram (**D**) of the effect of CPO (50 µg/ml) on the percentage of p21 expression levels in A549 cells. The results are expressed as the mean ± SD values from at least three independent experiments. ***P < 0.001 for the CPO-treated group in comparison to the control group

Due to cancer proliferative markers correlating variably based on the decoupled duration of cell cycle phases, cell cycle analysis and p21 expression levels were estimated after treatment with CPO. Cell cycles were analyzed with flow cytometry (P < 0.001; Fig. 2A and B) and the results showed a potent inhibitory effect of CPO on the cell cycle phase distribution in the treated A549 cells. CPO treatment significantly increased DNA accumulation in the S phase compared with the control group indicating the arrest of tumor cells. After treatment with CPO, cell arrest was accompanied by a significant elevation in the mean percentages of the apoptotic profiles and a significant decrease in the mean percentages of cells in the G2/M phase compared to the control group. Figure 2C and D show a significant increase (P < 0.001) in the percentage of p21 expression levels in the CPO treatment group compared to control cells.

### CPO induced DNA damage in A549 cells

**Fig. 3:**
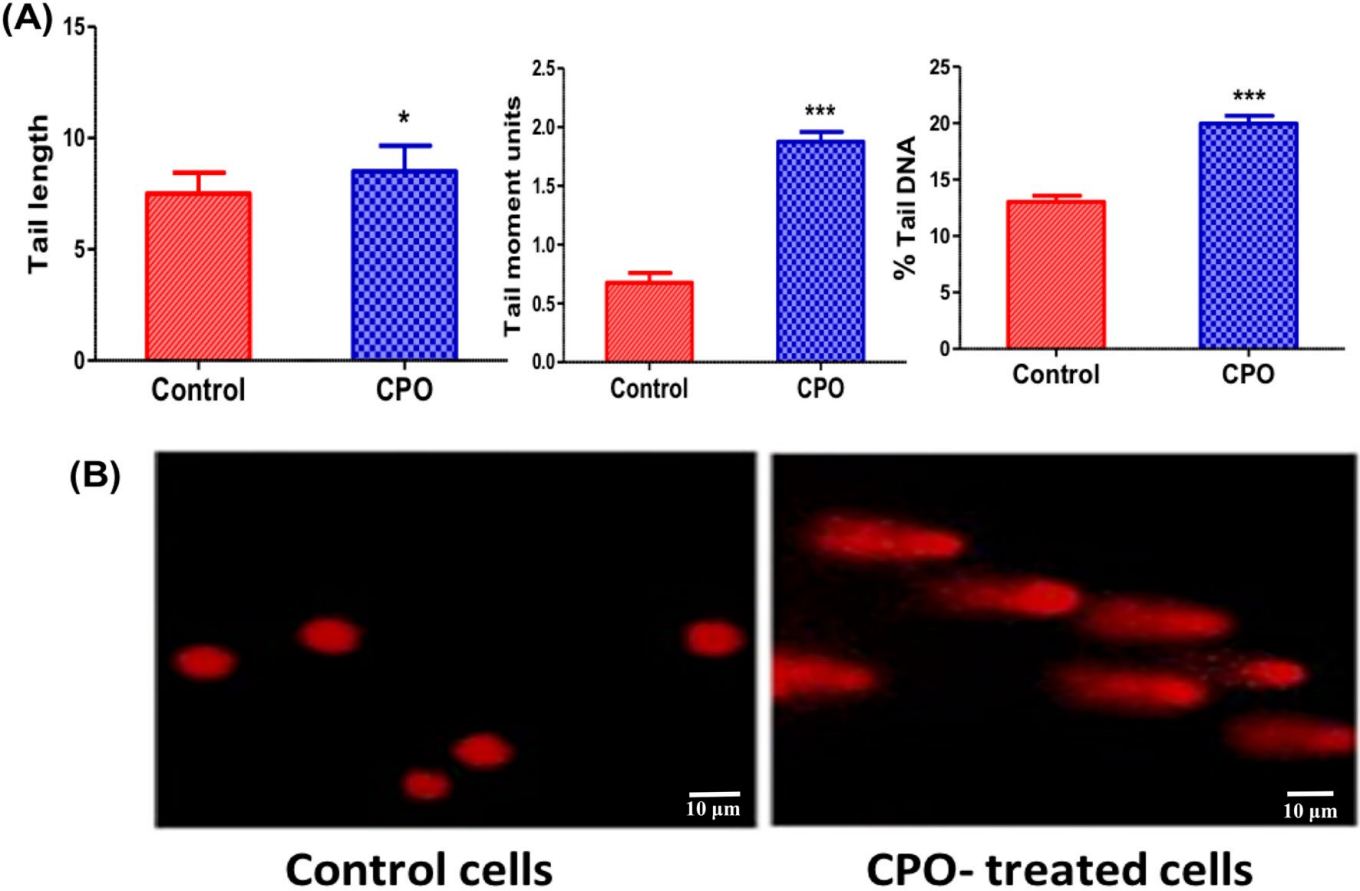
The effect of CPO (50 µg/ml) on DNA damage in the A549 lung cancer cell line after 24 h treatment. **A** Percentage of comet parameters (tail length, tail moment, and tail DNA) of DNA damage in A549 cells assessed by the comet assay; scale bar: 10 µm, and **B** Representative microscopic images of comets for the control cells and CPO-treated cells. The results are expressed as the mean ± SD values from at least three independent experiments. *P < 0.05 and ***P < 0.001 for the CPO-treated group in comparison to the control group

DNA damage was measured as a possible cause of the cell cycle arrest and apoptosis. A comet assay was conducted to evaluate the DNA strand breaks in A549 cells after CPO. Figure 3A and B showed a significant increase in tail length (P < 0.05), tail moment area (P < 0.001), and % tail DNA content (P < 0.001) compared to the untreated control cells.

### CPO caused apoptosis in A549 cells

**Fig. 4:**
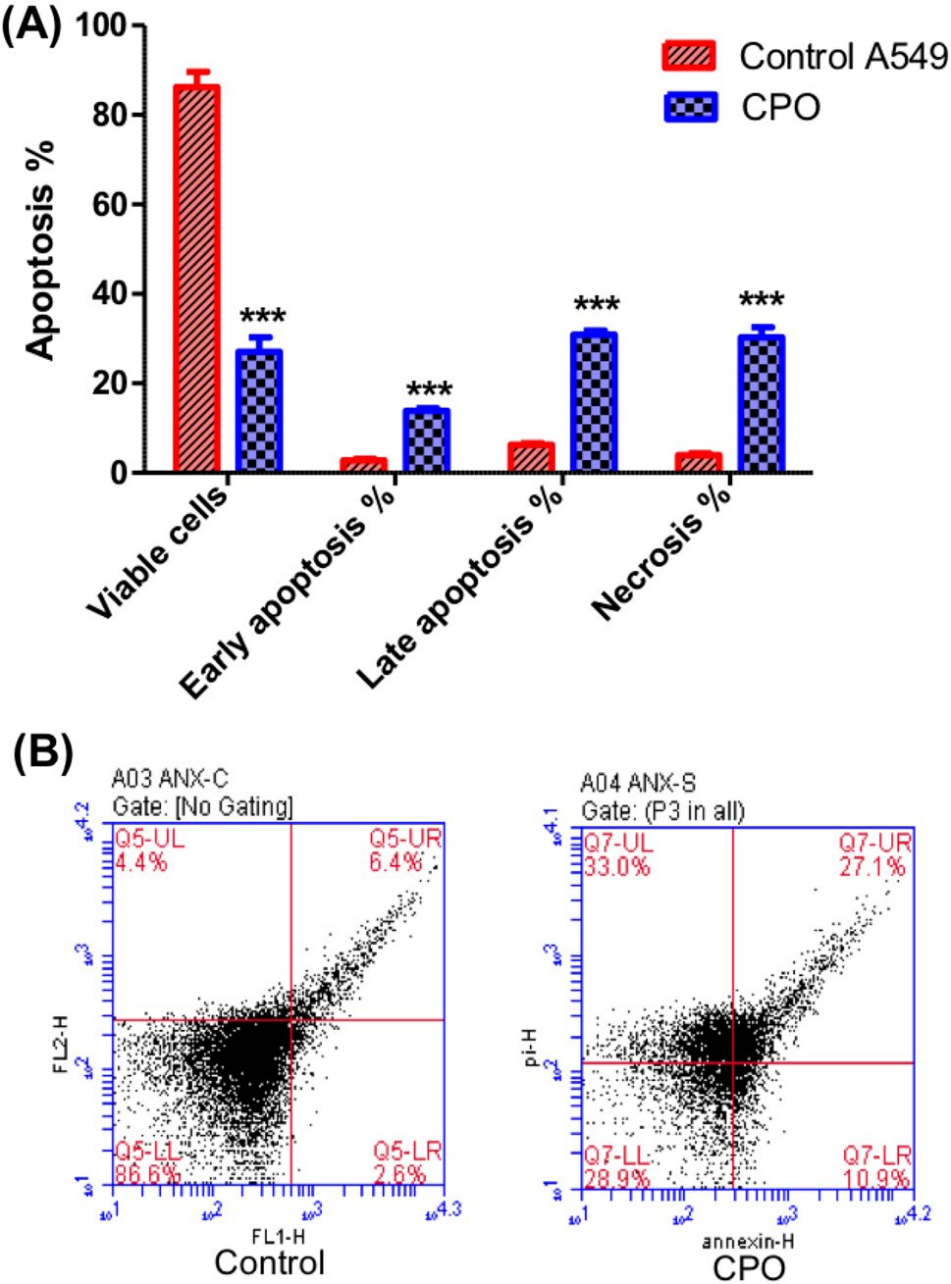
Flow cytometry analysis of apoptosis in the A549 cell line. Cells were treated with CPO for 24 h and then stained with Annexin V/PI. The results are expressed as the mean ± SD values from at least three independent experiments. ***P < 0.001 for the CPO (50 µg/ml) treated group in comparison to the control group

To examine the effects of CPO treatment on apoptosis of A549 cells, Annexin V-FITC and PI staining-flow cytometry was used after 24 h of treatment. The treatment of cells with CPO, at a dose of 50 μg/ml, induced marked apoptosis of the A549 cells. Regarding the apoptotic pattern of treated cells, there was a significant elevation in the percentage of early, late apoptotic cells and necrotic cells when cell treated with CPO in comparison with control cells (P < 0.001; Figs. 4A and B).

### CPO modulated the expression levels of apoptotic markers in A549 cells

**Fig. 5:**
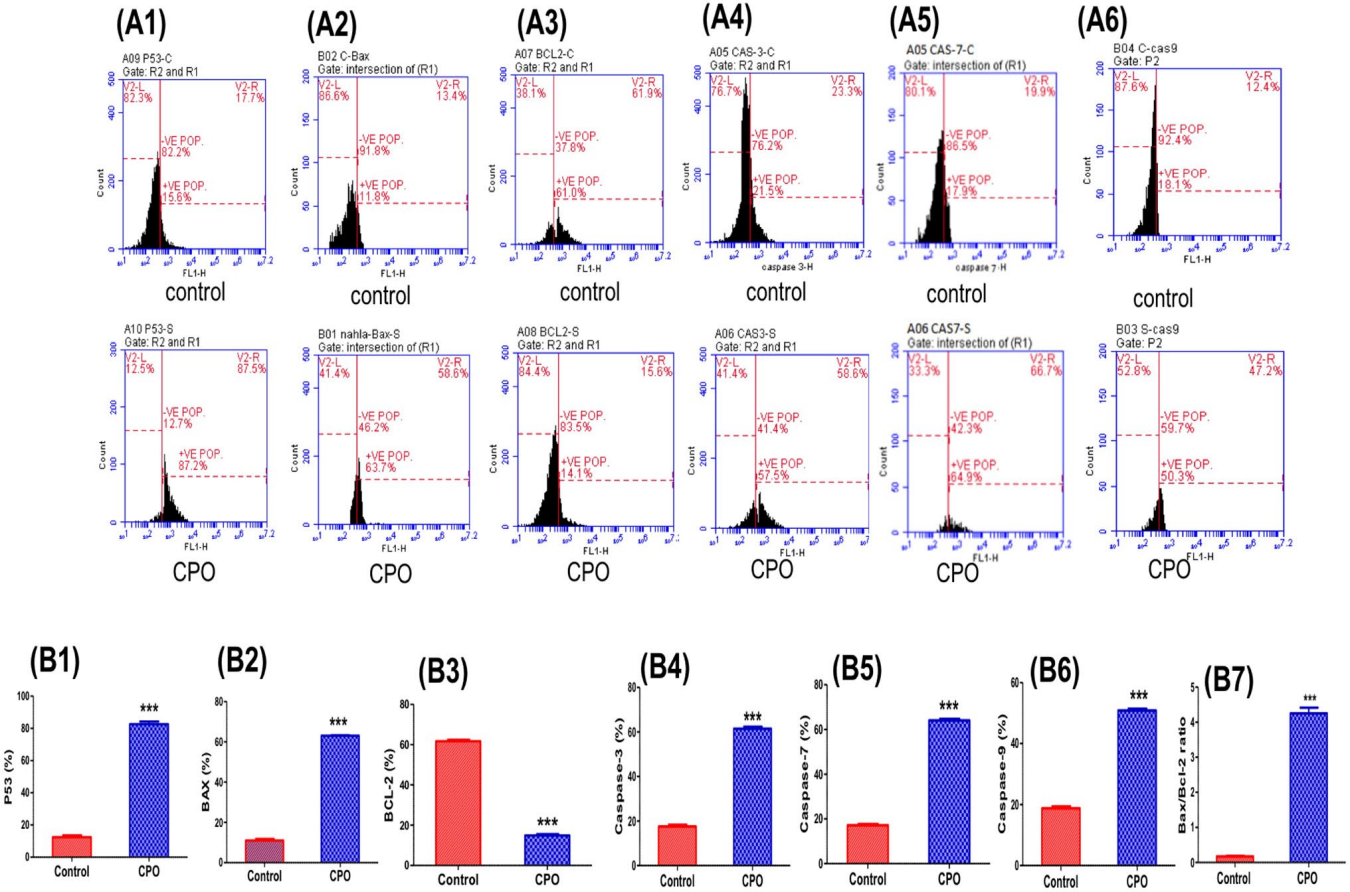
The effects of CPO (50 µg/ml) treatment for 24 h on the expression levels of apoptotic mediators in the A549 lung cancer cell line. (A) Flow cytometric histogram of apoptotic protein markers including P53 (A1), Bax (A2), Bcl-2 (A3), caspase-3 (A4), caspase-7 (A5), and caspase-9 (A6). (B) Percentage changes of P53 (B1), Bax (B2), Bcl-2 (B3), caspase-3 (B4), caspase-7 (B5), caspase-9 (B6), and the Bax/Bcl-2 ratio (B7). The results are expressed as the mean ± SD values from at least three independent experiments. ***P < 0.001 for the CPO-treated group in comparison to the control group

To identify the mechanism of the antiapototic effect of CPO, changes in the expression levels of related apoptotic-proteins were evaluated by flow cytometric analysis in lung cancer cells (A549). Treatment with CPO resulted in a significant upregulation in p53 expression compared to the control group (P < 0.001; Figs. 5A1 and 5B1). The effects of CPO treatment on protein expression levels of three important mediators of apoptosis, including caspase-3 (Figs. 5A4 and 5B4), caspase-7 (Figs. 5A5 and 5B5), and caspase-9 (Figs. 5A6 and 5B6), were also evaluated. Our results demonstrated that CPO significantly increased the expression of caspases-3, -7, and -9 in comparison to the control group (P < 0.001). In addition, FACS results showed that CPO resulted in a significant increase in the expression levels of pro-apoptotic protein and Bax (P < 0.001; Figures. 5A2 and 5B2) and a significant downregulation of the anti-apoptotic protein Bcl-2 (P < 0.001; Figs. 5A3 and 5B3). Additionally, the Bax/Bcl-2 ratio was significantly higher in the treated group than in the untreated group (P < 0.001).

### CPO increased the antioxidants and decreased lipid peroxidation in A549 cells

**Fig. 6:**
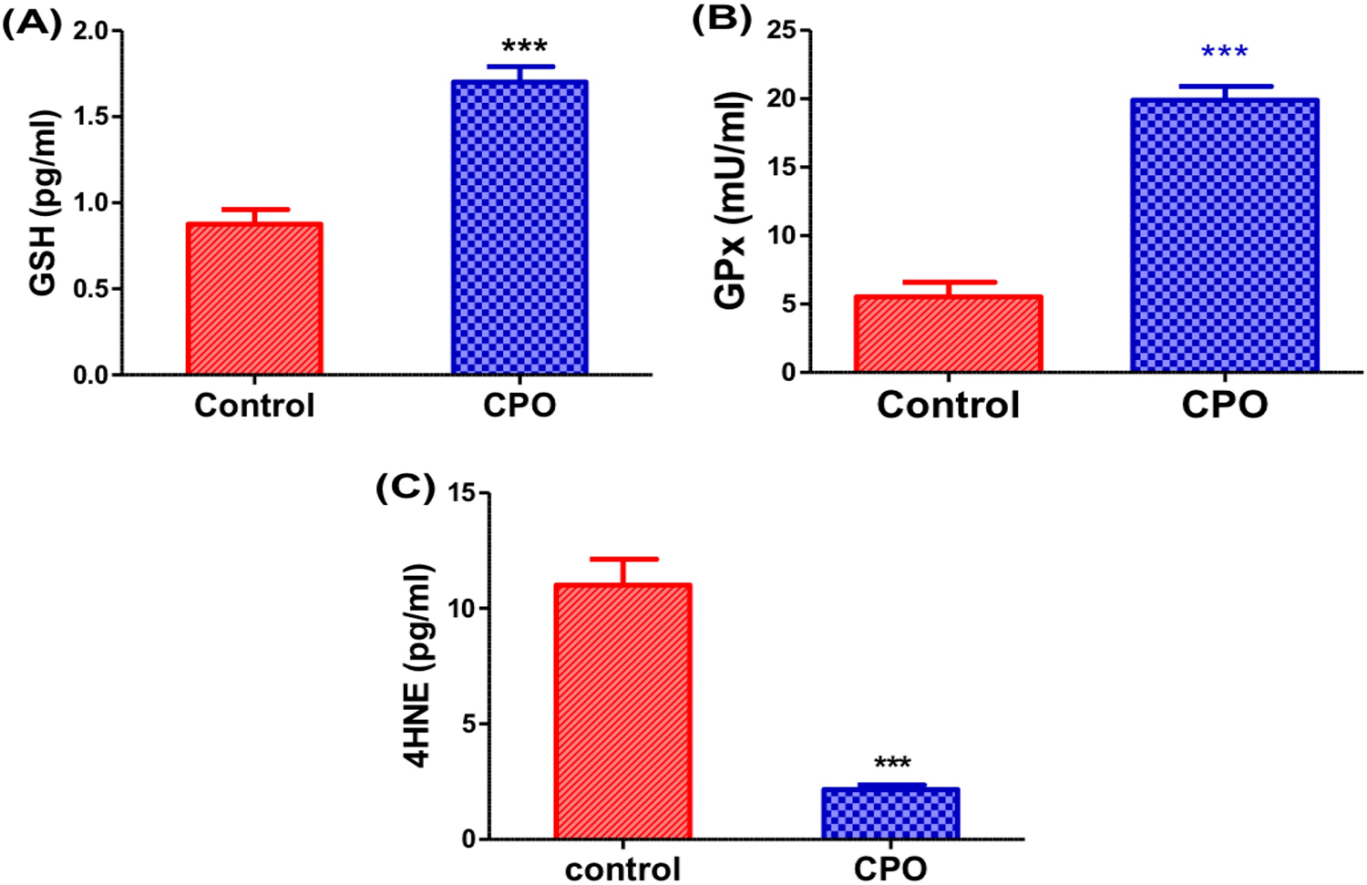
The effects of CPO (50 µg/ml) treatment for 24 h on antioxidants and oxidative stress markers. Expression levels of GSH (pg/ml) (**A**), GPX (mU/ml) (**B**) and 4HNE (pg/ml) (**C**) in the A549 cells. The results are expressed as the mean ± SD values from at least three independent experiments. ***P < 0.001 for the CPO-treated group in comparison to the control group. *CPO* β­caryophyllene oxide, *GSH* glutathione, *GPx* glutathione peroxidase, *4-HNE* 4-hydroxynonenal

The CPO-treated cells showed a significant increase in the enzymatic activity of GPx (P < 0.001; Fig. 6B) as well as highly significant elevation in levels of GSH (P < 0.001; Fig. 6A). A significant decrease in 4HNE (P < 0.001; Fig. 6C) levels was observed compared to the control cells.

## Discussion

This study investigated the inhibitory mechanism of CPO on NSCLC cells (i.e., A549 cells) by assessing apoptosis, apoptotic regulating proteins, cell cycle arrest, and the redox balance. There is more and more evidence that essential oils can inhibit the growth of cancer cells by targeting important signaling pathways that drive tumors [18]. Many studies have demonstrated that CPO has anti-proliferative effects on a variety of carcinoma cell lines [21]. The present study showed that CPO inhibited the proliferation and growth of A549 cells in vitro. This finding agreed with previous reports that showed CPO exhibited remarkable anticancer effects and inhibited growth and proliferation in several cancer cell types [12, 18].The present findings confirmed a previous report that CPO inhibited cell proliferation and attributed the inhibitory effect to a direct modulation of cannabinoid receptor 2 in glioblastoma cells [22].

Downregulation of the proliferative markers, including proliferating cell nuclear antigen (PCNA) and Ki67, confirmed a significant reduction in the proliferation of A549 cells. PCNA and Ki67 are cell cycle-specific antigens and prognostic proliferation standard markers for several types of cancer [3, 23]. The current data attributed the decrease in the viability of A549 cells after CPO treatment to the decrease in Ki-67 and PCNA expressions, signifying its chemotherapeutic effectiveness on lung cancer. This suggests that the important action of CPO might be inhibition of the expression of proteins that promote cell proliferation. Based on these results, a hypothesis for the anticancer action of CPO has been proposed. In a new study, researchers were able to demonstrate that CPO inhibited the growth of lung cancer using the A549 cells [24].

Typically, an uncontrolled cell cycle is a hallmark of cancer cells and this results in elevated cell proliferation [25]. Therefore, suppressing the cell cycle is an effective strategy for inhibiting cancer proliferation. The incubation of A549 cells with CPO for 24 h caused a significant increase in DNA accumulation in the S phase indicating cell cycle arrest. This suggested that CPO triggers defective cell cycles probably through stimulation of a checkpoint to arrest or eradicate such defective cells or it results in significant DNA injury that induces cell cycle arrest [26]. Moreover, the arrest of the cell cycle is supported by a significant decrease in the expression levels of the proliferation markers Ki 67and PCNA in CPO-treated A549 cells. The nuclear antigen Ki67 is expressed in the growth and synthesis phases of the cell cycle and then decreases at the end of M phase, while PCNA showed an increase in expression in the M to G1 phases [27], suggesting an anticancer indication. Consistent with this suggestion, the current study demonstrated that CPO was effective in inducing growth inhibition, cell cycle deregulation, and apoptosis in CPO cells. Cell cycle analysis showed that CPO induced S phase arrest in ovarian cancer cells (OAW 42 cells) [28]. These results suggest that CPO regulates the uncontrolled cell cycle in A549 cells. This information is crucial since tumors have uncontrolled cell cycles, and using CPO to target cell cycles could be an intriguing therapeutic strategy for malignancies, which confers a more powerful translational potential to CPO.

Apoptotic pathways are controlled by the balance between proteins that mediate decreased cell divisions with cell death, including p53, p21, and Bax, and proteins that promote cell viability, such as Bcl-2 [29]. Apoptosis is a type of cell death that is regulated by several proteins that are produced by specific genes. Apoptosis proceeds through activation of a programmed pathway of biochemical reactions that directly lead to cell death. Bax is a member of the Bcl-2 family and it functions to promote apoptosis and obstruct the cell survival effect of Bcl-2 [30]. Our results demonstrated that CPO-treated cells had considerably higher expression levels of apoptotic regulating proteins (P53, Bax, and caspase-3 and -7) than did untreated cells. The expression of the antiapoptotic protein Bcl-2 was concurrently significantly (P > 0.001) reduced in CPO-treated cells. In line with earlier research, it has been found that CPO inhibits tumour growth and induces apoptosis in human prostate and breast cancer cells by inhibiting PI3K/AKT/mTOR/S6K1 [31] and in non-small cell lung cancer by affecting miR-659-3p-targeted sphingosine kinase 1 [35]. Additionally, HeLa cells (human cervical adenocarcinoma cells), Hepatocellular cancer cells, gastric cancer cells (SNU-1), and stomach cancer cells (SNU-16) are all cytotoxic when exposed to CPO isolated from Jeju guava (*Psidium cattleianum Sabine*) leaves [15]. The hypothesis of apoptosis induction in our experimental conditions was supported by the elevated DNA damage estimated by a comet assay. These data were confirmed by the increase of p53, p21, Bax, and the major executioner caspases in the caspase cascade indicating the anticancer effects of CPO on A549. It has been reported that cysteine proteases, including the interleukin-1 beta-converting enzyme (ICE), are essential for the downstream events that control apoptosis in both nematodes and mammals [32]. Despite ROS being able to induce oxidative stress, they are not essential for apoptotic processes to occur [32]. ICE-family proteins are cellular apoptotic inducers [33], however, the signaling pathway has not been fully elucidated yet. Current study's results are reinforced by the interpretation that CPO, the most common ingredient, induced both early and late apoptosis in cancer cells. Additionally, it depolarized the mitochondrial membrane, resulting in numerous alterations to the form and localization of apoptotic proteins. The pro-apoptotic protein Bax is liberated from Bcl-xL by the depolarization of the mitochondrial membrane. The activation of caspase-7 is essential for the apoptosis process [34]. These results suggest that CPO has the potential to activate the intrinsic signaling apoptotic pathway and inhibit cell growth and proliferation; however, the mechanism for induction of apoptosis by CPO remains to be identified.

Cancer cells are characterized by increased mitochondrial activity and excessive production of ROS, which promote cancer proliferation and growth [35]. Thus, reduction of mitochondrial ROS production by antioxidants is a plausible approach to protect mitochondria from oxidative processes [5]. The present results showed that CPO increased antioxidants (GSH and GPx) and decreased lipid peroxidation (4HNE) in A549 cells, which is associated with the inhibition of the proliferation of lung cancer cells. These results are consistent with earlier reports that showed a marked reduction of ROS generation and increased antioxidant levels in A549 cells after CPO treatment [36, 37], an effect that was associated with inhibition of cancer cell proliferation. Moreover, in an in vivo study, treatment with Wedelia chinensis essential oil extracts, 96% of which are carvacrol and caryophyllene, increases intracellular antioxidant activity, which in turn causes a considerable decrease in the mass volumes of tumors and the regeneration of healthy tissue around them [38]. The radical scavenging capability of CPO is well documented [37]. Thus, the current study also suggests that the anticancer effect of CPO is independent of the increase in oxidative stress that can induce cancer cell death and the intrinsic pathway is not activated by ROS in A549 cells treated with CPO. This is consistent with the suppression of ROS-mediated mitogen-activated protein kinase activation by CPO in lung cancer cells [31], which inhibits cancer progression. As a result, CPO might be used as an alternate treatment agent for preventing metastasis.

## Conclusions

In conclusion, molecular effectors, which influence cell cycle activity, proliferation, and apoptotic pathways, are involved in mediating the anticancer effects of CPO. Additionally, the mechanism for inhibition of proliferation was suggested to be independent of oxidative stress and promoted with antioxidants. More research is warranted on the chemopreventive and possible sensitization effects of CPO therapy or co-treatment with therapeutic medications in order to explore CPO as a viable adjuvant therapeutic. To confirm the advantages of CPO’s therapeutic use, study of its anticancer activities using various cancer models is also necessary. CPO reactions with the cannabinoid receptor 2 in A549 lung cells should be explored.

## Data Availability

The authors confirm that the data and materials supporting the findings of this study are available within the article.
